# Protective effects of baicalin on rabbit articular chondrocytes *in vitro*

**DOI:** 10.3892/etm.2021.9768

**Published:** 2021-02-08

**Authors:** Xianyuan Huang, Huayu Wu, Liqin Wang, Li Zheng, Jinmin Zhao

Exp Ther Med 13:1267–1274, 2017; DOI: 10.3892/etm.2017.4116

Subsequently to the publication of the above article, the authors have realized that both Figs. 3 and 5 contained some data panels that were inadvertently selected erroneously. Specifically, in Fig. 3, the 0.625 *μ*mol/l and 2.5 *μ*mol/l panels for the Day 4 data were selected incorrectly, whereas the Control panels for the Day 2 and Day 4 experiments, and also the 0.625 *μ*mol/l Day 4 panel, in Fig. 5 were shown incorrectly.

The revised versions of Figs. 3 and 5 containing the corrected data are shown on the next page. All the authors agree to this Corrigendum; also note that these errors did not affect the overall conclusions reported in the paper. The authors regret these errors, and apologize for any confusion that these mistakes have caused.

## Figures and Tables

**Figure 3:**
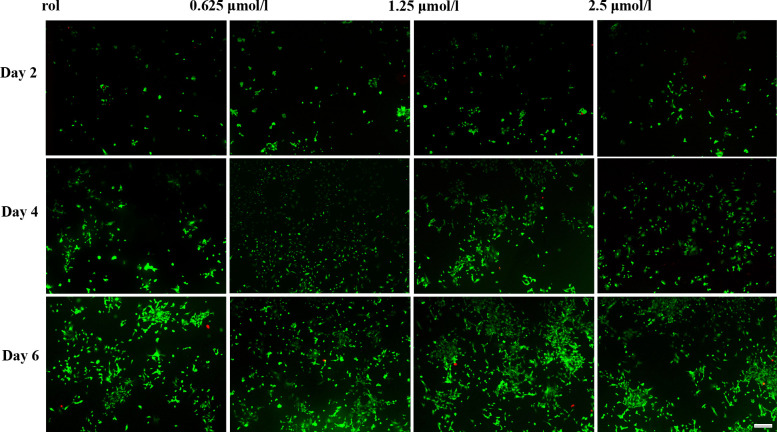
Fluorescein diacetate/propidium iodide staining images indicating the live (green) and dead (red) chondrocytes cultured *in vitro* with 0 (control), 0.625, 1.25 and 2.5 μmol/l baicalin for 2, 4 and 6 days (magnification, ×40; scale bar, 200 μm).

**Figure 5:**
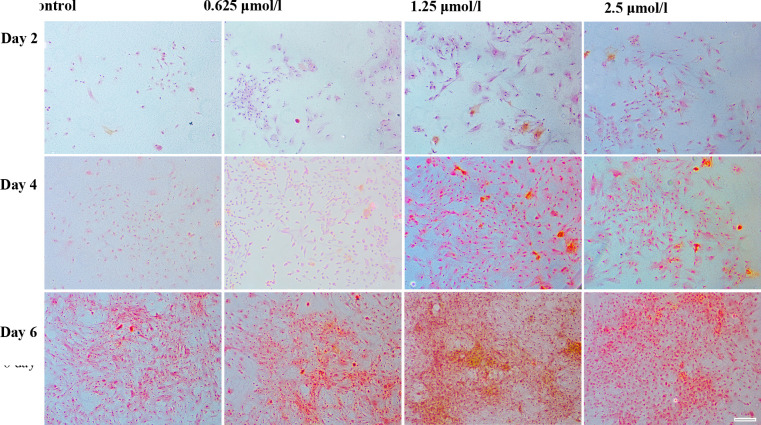
Safranin O staining demonstrating *in vitro* extracellular matrix synthesis by chondrocytes (seeding density, 2×10^4^/ml) cultured with 0 (Control), 0.625, 1.25 and 2.5 μmol/l baicalin for 2, 4 and 6 days (magnification, ×200; scale bar, 100 μm).

